# Two-Year Outcomes of Umbilical Cord Milking in Nonvigorous Infants: A Secondary Analysis of the MINVI Randomized Clinical Trial

**DOI:** 10.1001/jamanetworkopen.2024.16870

**Published:** 2024-07-01

**Authors:** Anup C. Katheria, Laure El Ghormli, Erin Clark, Bradley Yoder, Georg M. Schmölzer, Brenda H. Y. Law, Walid El-Naggar, David Rittenberg, Sheetal Sheth, Courtney Martin, Farha Vora, Satyan Lakshminrusimha, Mark Underwood, Jan Mazela, Joseph Kaempf, Mark Tomlinson, Yvonne Gollin, Wade Rich, Ana Morales, Michael Varner, Debra Poeltler, Yvonne Vaucher, Judith Mercer, Neil Finer, Madeline Murguia Rice

**Affiliations:** 1 Neonatal Research Institute, Sharp Mary Birch Hospital for Women & Newborns, San Diego, California; 2 George Washington University Biostatistics Center, Milken Institute School of Public Health, Rockville, Maryland; 3 School of Medicine, University of Utah Salt Lake City; 4 Faculty of Medicine and Dentistry, University of Alberta Alberta, Canada; 5 Dalhousie University, Halifax, Nova Scotia, Canada; 6 School of Medicine, George Washington University, Washington, DC; 7 Loma Linda University, Loma Linda, California; 8 School of Medicine, University of California, Davis, Sacramento; 9 Poznan University of Medical Sciences, Poznan, Poland; 10 Providence St Vincent Medical Center, Portland, Oregon; 11 University of California, San Diego; 12 University of Rhode Island, Kingston

## Abstract

**Importance:**

Compared with early cord clamping (ECC), umbilical cord milking (UCM) reduces delivery room cardiorespiratory support, hypoxic-ischemic encephalopathy, and therapeutic hypothermia in nonvigorous near-term and full-term infants. However, UCM postdischarge outcomes are not known.

**Objective:**

To determine the 2-year outcomes of children randomized to UCM or ECC at birth in the Milking in Nonvigorous Infants (MINVI) trial.

**Design, Setting, and Participants:**

A secondary analysis to evaluate longer-term outcomes of a cluster-randomized crossover trial was conducted from January 9, 2021, to September 25, 2023. The primary trial took place in 10 medical centers in the US, Canada, and Poland from January 5, 2019, to June 1, 2021, and hypothesized that UCM would reduce admission to the neonatal intensive care unit compared with ECC; follow-up concluded September 26, 2023. The population included near-term and full-term infants aged 35 to 42 weeks’ gestation at birth who were nonvigorous; families provided consent to complete developmental screening questionnaires through age 2 years.

**Intervention:**

UCM and ECC.

**Main Outcomes and Measures:**

Ages and Stages Questionnaire, 3rd Edition (ASQ-3) and Modified Checklist for Autism in Toddlers, Revised/Follow-Up (M-CHAT-R/F) questionnaires at ages 22 to 26 months. Intention-to-treat analysis and per-protocol analyses were used.

**Results:**

Among 1730 newborns from the primary trial, long-term outcomes were evaluated in 971 children (81%) who had ASQ-3 scores available at 2 years or died before age 2 years and 927 children (77%) who had M-CHAT-R/F scores or died before age 2 years. Maternal and neonatal characteristics by treatment group were similar, with median birth gestational age of 39 (IQR, 38-40) weeks in both groups; 224 infants (45%) in the UCM group and 201 (43%) in the ECC group were female. The median ASQ-3 total scores were similar (UCM: 255 [IQR, 225-280] vs ECC: 255 [IQR, 230-280]; *P* = .87), with no significant differences in the ASQ-3 subdomains. Medium- to high-risk M-CHAT-R/F scores were also similar (UCM, 9% [45 of 486] vs ECC, 8% [37 of 441]; *P* = .86).

**Conclusions and Relevance:**

In this secondary analysis of a randomized clinical trial among late near-term and full-term infants who were nonvigorous at birth, ASQ-3 scores at age 2 years were not significantly different between the UCM and ECC groups. Combined with previously reported important short-term benefits, this follow-up study suggests UCM is a feasible, no-cost intervention without longer-term neurodevelopmental risks of cord milking in nonvigorous near-term and term newborns.

**Trial Registration:**

ClinicalTrials.gov Identifier: NCT03631940

## Key Points

**Question:**

Among nonvigorous term and near-term newborns, does umbilical cord milking compared with early cord clamping at birth result in improved neurodevelopmental outcomes?

**Findings:**

In this secondary analysis to evaluate longer-term outcomes of a randomized clinical trial in 971 infants, the differences in the Ages & Stages Questionnaire total scores between the early cord clamping vs the umbilical cord milking group were not statistically significant. Differences in the medium- to high-risk scores on the Modified Checklist for Autism in Toddlers, Revised/Follow-Up, between the 2 groups were also not statistically significant.

**Meaning:**

The findings of this trial suggest that, among nonvigorous term and near-term infants, umbilical cord milking does not result in a statistically significant difference in 2-year neurodevelopmental outcomes compared with early cord clamping.

## Teaser

This secondary analysis of a randomized clinical trial compares neurodevelopmental outcomes after the use of umbilical cord milking or early cord clamping in near-term or term nonvigorous infants at age 2 years.

## Introduction

Guidelines^1,2^ and randomized clinical trials^3^ have demonstrated the benefits of delaying umbilical cord clamping at birth at least 30 to 60 seconds, which enhances newborn blood volume. Delayed cord clamping may improve neurodevelopmental outcomes in full-term infants^4^ and survival in preterm infants.^5^ Optimal umbilical cord management in nonvigorous (limp, pale, and minimal or no breathing) infants who may need resuscitation after birth is unclear.^2,3^ Our study group^6^ conducted the Milking in Nonvigorous Infants (MINVI) trial, which provided, to our knowledge, the largest amount of randomized clinical trial to date in nonvigorous near-term and full-term infants comparing umbilical cord milking (UCM) with early cord clamping (ECC). In that study, after a brief assessment of the infant’s color, tone, and breathing was performed in the first 10 to 15 seconds of life, infants assessed as nonvigorous received UCM (milking of 20 cm of cord over 2 seconds a total of 4 times) or ECC (cord clamped within 60 seconds of birth). The primary outcome of admission to the neonatal intensive care unit was not significantly different between groups.^7^ However, secondary outcomes of delivery room cardiorespiratory support, hypoxic-ischemic encephalopathy, and therapeutic hypothermia were lower in infants randomized to UCM.^7^ Umbilical cord milking enhances blood volume similarly to delayed UCC^7,8^ but has the advantage of allowing resuscitation to begin sooner.

Long-term risks of UCM in near-term and full-term infants are unknown. Data from a anesthetized preterm lamb model suggest UCM results in fluctuations in carotid artery blood flow,^9^ which dissipates if the lambs are ventilated.^10,11^ A randomized clinical trial stopped enrollment in the most preterm strata (23-27 weeks’ gestation) due to an associated increase in severe intraventricular hemorrhage.^12^ There were no significant differences in short-term outcomes in the more mature group (28-32 weeks’ gestation).^13^ Three trials in preterm infants did not show significant differences in neurodevelopmental problems comparing UCM with ECC or delayed cord clamping.^14,15,16^ All 3 trials were underpowered to assess neurodevelopmental risk or benefit. It remains unclear whether the rapid infusion of blood (UCM) might be associated with long-term neurodevelopmental sequalae in near-term and/or full-term infants.

## Methods

We conducted a preplanned secondary analysis (January 9, 2021, to September 25, 2023) of participants whose parents provided informed consent in the primary randomized clinical trial (January 5, 2019, to June 1, 2021) to evaluate the rates of abnormal developmental scores on the Ages and Stages Questionnaire (ASQ)^17^ and rates of medium- to high-risk scores on the Modified Checklist for Autism in Toddlers, Revised/Follow-Up (M-CHAT-R/F)^18^ at 2 years of age and compared the rates between the 2 intervention groups. Follow-up concluded September 26, 2023. The research ethics board or institutional review board at participating sites approved a waiver of antenatal consent to implement the protocols and collect data on death before hospital discharge on potential participants in the primary trial. This study followed the Consolidated Standards of Reporting Trials (CONSORT) reporting guideline.

This was a pragmatic (resembling real-world settings with minimal exclusions and a group or a cluster randomization),^19^ cluster-randomized crossover trial of nonvigorous infants born at 35 to 42 weeks’ gestation in 10 medical centers randomized to either UCM or ECC protocols, then crossed over to the other treatment condition after 1 year of trial participation (protocol and statistical analysis plan are available in Supplement 1). Infants were eligible for enrollment in the primary trial if nonvigorous (poor tone, pale color, or lack of breathing in the first 15 seconds after birth). Infants were excluded if the following conditions were known prior to cord clamping: major fetal congenital or chromosomal anomalies; cardiac defects other than small ventricular septal defects; complete placental abruption or cutting through the placenta at the time of delivery; monochorionic multiples; cord anomalies, such as avulsion or true knots; presence of a nonreducible nuchal cord; and incomplete delivery data to determine eligibility. Infants were then assigned to UCM (milking the attached cord 4 times in 20 seconds) or ECC (clamping the cord before 60 seconds).^7^ Collection and reporting of race and ethnicity were required by the National Institutes of Health and self-reported by parents at the time of contact for informed consent to the long-term follow-up portion of the trial. After delivery, families were asked to consent to long-term neurodevelopmental follow-up of the infant using the ASQ, 3rd Edition (ASQ-3),^20^ and risk assessment for autism using the M-CHAT-R/F. Signed parental consent was required for participation in the long-term developmental follow-up phase of the study. Each site had a designated follow-up team that maintained contact with the child’s family to facilitate completion of ASQ-3 at ages 6, 12, and 22 to 26 months, and M-CHAT-R/F at 22 to 26 months. The follow-up team provided feedback to the family regarding developmental screening results and referred children to appropriate resources if major problems were identified. To enhance follow-up, each site maintained ongoing contact with families by text, phone, email, and/or written communication, with a goal of at least 80% questionnaire completion.

The ASQ-3 is a parent-reported, developmental screening tool used worldwide for children aged 2 to 60 months that covers 5 developmental subscales (communication, problem-solving, fine motor, gross motor, and personal-social).^17^ Each scale consists of 6 items assessed to a possible 60 points, with a maximum total score of 300 points. Items are reported by parents who rate each behavior as not yet (0 points), sometimes (5 points), or yes (10 points). Higher scores indicate more developmental milestones reached. Cutoff scores are created according to the ASQ-3 manual. Scores less than 2 SDs below the mean of each domain are considered subnormal. Infants who died before or after discharge but before completion of the questionnaires were by default assigned an ASQ-3 score of 0.

The parent or caregiver completed the questionnaire with assistance from the follow-up team. All parent questionnaires were offered via ASQ online.^21^ If a family preferred not to use internet or did not have internet access, paper copies were mailed. Survey results and feedback were provided to the parents by the follow-up team. If a problem was identified, participants were referred to their pediatrician or the site’s High-Risk Infant Follow-Up Clinic for further evaluation. All surveys were published in both English and Spanish.

The M-CHAT-R/F is a parent-reported tool used to screen for autism spectrum disorder (ASD).^18^ It is designed to maximize sensitivity for autism detection but has an elevated false-positive rate.^18,22^ The 20-item questionnaire asks for yes or no answers. If the total score is 0 to 2, the child is considered low risk, 3 to 7 is considered medium risk, and 8 to 20 indicates high risk for ASD.

### Statistical Analysis

Descriptive statistics were used, with the Wilcoxon rank-sum test for continuous variables and the χ^2^ test for categorical variables. Data on infants with consent for long-term follow-up (alive or dead) were analyzed according to intention to treat, and all analyses were prespecified in the trial protocol. Differences in ASQ-3 and M-CHAT-R/F scores between treatment groups were evaluated at age 2 years (primary analyses) and at 6 and 12 months (secondary analyses, ASQ-3 only). The study was designed to provide at least 80% power to detect a small effect size (Cohen *d* = 0.2) for between-treatment group difference in ASQ-3 scores under a type I error, with α = .05, assuming 20% missing data at age 2 years. Analyses were conducted using hierarchical generalized linear mixed models accounting for the cluster-randomized crossover study design, with fixed treatment group effect, fixed period effect, random cluster effect, and random cluster × period effect. All models also included adjustment for child sex and maternal educational level (2 predictors of child neurodevelopment). Least-squares mean differences for continuous outcomes and odds ratios for categorical outcomes, along with 95% CIs, were estimated. Two-sided *P* values <.05 were considered to indicate statistical significance. Analyses were performed with the use of SAS software, version 9.4 (SAS Institute Inc).

## Results

At age 2 years, 971 participants (81%) had ASQ-3 scores completed or had died. There were no major differences by treatment group in maternal and neonatal characteristics, with median birth age of 39 (IQR, 38-40) weeks’ gestational age in both groups; 224 (45%) infants in the UCM group were female and 278 (55%) were male, and 201 (43%) in the ECC group were female and 268 (57%) were male. Most children included in the analysis had mothers of non-Hispanic ethnicity 363 (73%) in the UCM group and 333 (71%) in the ECC group. Race and ethnicity was self-reported as unknown or not reported in 53 (11%) infants in the UCM group and 48 (10%) in the ECC group (Table 1). A total of 486 infants (97%) were treated per protocol in the UCM group, and 465 (99%) in the ECC group (Table 1). There was no significant difference by treatment group in neonatal intensive care unit admission for predefined criteria as previously reported between the 2 groups^3^ (eTable 1 in Supplement 2). Among the 1730 nonvigorous newborns enrolled in the primary trial, 1206 were discharged alive and the parents provided consent for long-term follow-up, 4 infants died in the hospital, and 3 infants died after discharge (Figure). Of the 1730 newborns enrolled, the 971 infants included in the analysis were more likely to have been born by cesarean delivery, have older mothers, and a higher maternal educational level compared with the 759 infants not evaluated for neurodevelopment (eTable 2 in Supplement 2). All other maternal and neonatal characteristics were similar between those included in the analysis vs those excluded. Similar results were observed for the 927 infants included in the M-CHAT-R/F analysis (77% of those consented).

**Table 1.** Maternal and Neonatal Baseline Characteristics by Randomization Group Among the 971 Infants With ASQ-3 Data Available for Analysis at 2 Years of Age

Characteristic	No. (%)^a^	
	Umbilical cord milking (n = 502)	Early cord clamping (n = 469)
Maternal		
Race and ethnicity^b^		
Non-Hispanic Asian	39 (8)	45 (10)
Non-Hispanic Black	34 (7)	34 (7)
Hispanic	86 (17)	88 (19)
Non-Hispanic White	290 (58)	254 (54)
Other/unknown	53 (11)	48 (10)
Age, mean (SD), y	31 (5)	31 (6)
At least some college education	349 of 446 (78)	321 of 406 (79)
Any diabetes	72 (14)	63 (13)
Any hypertension	89 (18)	81 (17)
Intrauterine inflammation or infection	54 (11)	62 (13)
GBS positive	114 of 501 (23)	88 of 469 (19)
Rupture of membranes before delivery, median (IQR), h	6 (1-14)	5 (1-14)
Narcotic/CNS depressant ≤2 h predelivery^c^	51 (10)	51 (11)
General anesthesia	32 of 502 (6)	33 of 468 (7)
Neonatal		
Female sex	224 (45)	201 (43)
Male sex	278 (55)	268
Cesarean mode of delivery	246 (49)	206 (44)
Multiple gestation	13 of 501 (3)	18 of 467 (4)
Gestational age at delivery, median (IQR), wk	39 (38-40)	39 (38-40)
Treated per protocol	486 (97)	465 (99)
Death before 2 y	2 (<1)	5 (1)

Abbreviations: ASQ-3, Ages and Stages Questionnaire, 3rd Edition; CNS, central nervous system; GBS, group B *Streptococcus*.

^a^ Denominators for all variables are as per column headers unless otherwise noted.

^b^ Other/unknown race, race not reported, or self-reported race not included in categorical choices.

^c^ Oral or intravenous administration.

**Figure.**
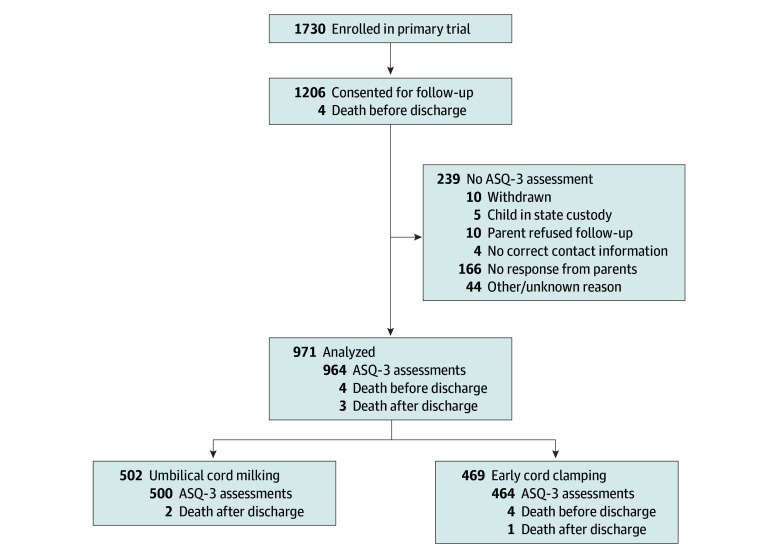
Flow Diagram of Secondary Analysis Population Ages and Stages Questionnaire (ASQ-3) data available for analysis at the age 22- to 26-month time point. Of the 971 newborns with data analyzed, a total of 927 newborns also had Modified Checklist for Autism in Toddlers, Revised/inFollow-Up data collected.

Median ASQ-3 total scores (UCM: 255 [225-280] vs ECC 255 [230-280]; *P* = .87; difference, −0.49; 95% CI, −6.36 to 5.37), any of the 5 subdomains, and medium to high-risk M-CHAT-R/F scores (UCM, 9% [45 of 486] vs ECC, 8% [37 of 441]; *P* = .86; OR, 1.08; 95% CI, 0.45-2.59) were all similar between treatment groups (Table 2). No significant difference by treatment group was observed for the ASQ-3 total and domain scores in infants evaluated at 6 and 12 months of age (eTable 3 in Supplement 2). Across the 5 subdomains, 4% to 11% of the children were identified to have severe delay (score ≥2 SD below the mean), with no significant difference by treatment group (Table 2). Overall, 78% of the UCM group vs 83% of the ECC group had normal scores in each of the 5 developmental subdomains (95% CI, 0.54-1.08; *P* = .12). The sex interaction was conducted but did not change the primary outcome.

**Table 2.** Developmental Screening Characteristics Assessed at 2 Years by Randomization Group, Including the 7 Infant Deaths (Weighted as 0)

Characteristic	Umbilical cord milking (n = 502)	Early cord clamping (n = 469)	Mean difference (95% CI)^a^
**Overall with ASQ-3 data (n = 971)**			
ASQ-3 domains, median (IQR)			
Communication	55 (40-60)	55 (40-60)	Least squares, 0.01 (−2.07 to 2.08)
Gross motor	60 (50-60)	60 (50-60)	Least squares, 0.08 (−3.25 to 3.42)
Fine motor	50 (45-60)	50 (45-60)	Least squares, 0.51 (−0.88 to 1.90)
Problem solving	50 (40-55)	50 (40-60)	Least squares, −0.19 (−1.70 to 1.32)
Personal-social	50 (40-55)	50 (45-55)	Least squares, −0.72 (−3.54 to 2.11)
Overall score	255 (225-280)	255 (230-280)	Least squares, −0.49 (−6.36 to 5.37)
**Infants with subnormal ASQ-3 scores** ^b^			
ASQ-3 domains, No. (%)			
Communication	54 (11)	51 (11)	OR, 0.96 (0.61 to 1.51)
Gross motor	33 (7)	25 (5)	OR, 1.38 (0.73 to 2.58)
Fine motor	25 (5)	20 (4)	OR, 1.44 (0.54 to 3.84)
Problem solving	29 (6)	26 (6)	OR, 1.18 (0.61 to 2.25)
Personal-social	47 (9)	35 (8)	OR, 1.33 (0.79 to 2.25)
All ASQ-3 domains indicate typical development	393 (78)	389 (83)	OR, 0.76 (0.54 to 1.08)
**Overall with M-CHAT-R/F data (n = 927)^c^**			
M-CHAT-R/F, No. (%)			
Score 0-2	441 (91)	404 (92)	NA
Score 3-7	34 (7)	31 (7)	OR, 1.06 (0.33 to 3.36)
Score 8-20	11 (2)	6 (1)	OR, 1.19 (0.41 to 3.50)
Score 0-2	441 (91)	404 (92)	NA
Score >2	45 (9)	37 (8)	OR, 1.08 (0.45 to 2.59)

Abbreviations: ASQ-3, Ages and Stages Questionnaire, 3rd Edition; M-CHAT-R/F, Modified Checklist for Autism in Toddlers, Revised/Follow-Up; NA, not applicable; OR, odds ratio.

^a^ Least-squares mean differences (for continuous scores) and ORs (for categorical scores) with corresponding 95% CIs are derived from models accounting for the clustered-crossover study and adjusted for infant sex and maternal education. Score 0 to 2 is used as the reference group in the M-CHAT-R/F model.

^b^ Defined as No. (%) with score greater than or equal to 2 SDs below the mean in at least 1 domain or death before age 2 years.

^c^ For umbilical cord milking, n = 486, and for early cord clamping n = 441.

## Discussion

In near-term and full-term infants nonvigorous at birth, UCM did not improve or worsen ASQ-3 scores compared with ECC. To our knowledge, this report is the largest to date of 2-year neurodevelopment parental assessments for a cord-milking trial. While the primary outcome, neonatal intensive care unit admission rate, was not significantly different when adjusted by center, prespecified short-term secondary outcomes were favorable in the UCM group: lower receipt of cardiorespiratory support, less development of hypoxic-ischemic encephalopathy and hypothermia treatment, and higher hemoglobin levels.^6^ Our present study concurs with previous reports of placental transfusion trials that did not show significant differences in long-term neurodevelopmental outcomes despite the short-term gains identified in the initial studies.^23,24^

Our short-term outcomes that favor UCM may provide confirmation of utility to practitioners with understandable reservations about placental transfusion in nonvigorous newborns. Recommendations from the American College of Obstetricians and Gynecologists do not recommend UCM in infants less than 28 weeks’ gestation based on the PREMOD2 trial, but acknowledge there is insufficient evidence to support or refute UCM in infants born at 32 weeks’ gestation or more.^2^ The report states, “infants requiring resuscitation may benefit considerably from placental transfusion, but their need for immediate attention raises questions about whether they should undergo immediate or delayed umbilical cord clamping and whether umbilical cord milking may offer a unique benefit.”^2^ The American Heart Association and American Academy of Pediatrics 2023 neonatal resuscitation guidelines states, “for nonvigorous term and late preterm newborn infants 35 to 42 weeks’ gestation, intact cord milking may be reasonable compared with early cord clamping.”^3^ These recommendations were based on the short-term benefits of UCM, conceivably related to enhanced blood volumes.^3^

### Strengths and Limitations

Strengths of our study include the randomized multicenter design with crossover to reduce intervention bias, the large population sample size from diverse centers in North America and Europe, excellent adherence to the study protocol, the lack of safety issues identified, and a reasonably high follow-up rate for neurodevelopmental assessment.

There are several limitations to our cluster-randomized crossover trial developmental follow-up assessment. A more comprehensive in-person evaluation to determine motor, language, and cognition (such as a Bayley Scales of Infant and Toddler Development) might have increased the sensitivity of detecting subtle differences between the 2 groups. Since this trial consisted of many full-term infants who did not require neonatal intensive care unit admission, we believed parents of healthy near-term or term newborns would be less willing or unable to have their child undergo in-person evaluations, so we opted for a pragmatic online questionnaire to facilitate a minimum 80% follow-up rate. The ASQ-3 has been shown to have good internal consistency and validity. Compared with the standard Bayley Scales of Infant and Toddler Development II, sensitivity of the ASQ-3 is moderate (78%) for any delay and very high (92%-100%) for severe delay.^25^ Compared with the more recently published Bayley Scales of Infant and Toddler Development III, the ASQ-3 questionnaire has excellent negative predictive value (98%), with subscale specificities ranging from 92% to 96%.^26^ The M-CHAT-R/F tool can be administered as part of a well-child care visit, and also can be used by other professionals to assess the risk for ASD. The primary goal of the M-CHAT-R/F is maximal sensitivity to detect as many cases of ASD as possible. There is a high false-positive rate, meaning not all children who score at risk will be diagnosed with ASD. Although a substantial number of children who screen positive on the M-CHAT-R/F will not be diagnosed with ASD, they are at high risk for other developmental disorders. Children with total scores greater than or equal to 2 have a 95% risk of subsequent developmental delay concerns (95% CI, 0.92-0.98).^27^

A second limitation is our investigation was conducted in relatively high-income academic centers. Most women had sufficient prenatal care, and almost 80% of the mothers had some college education. Whether our encouraging findings are applicable to lower resource centers remains to be determined and is crucial because UCM is a facile, 0-cost intervention.

In this secondary analysis of a randomized clinical trial, the incidence of severe morbidity, such as hypoxic-ischemic encephalopathy and death, was low, and 73% of the infants did not require admission to the neonatal intensive care unit. Our results may not be translatable to populations of infants at high risk for morbidity and mortality, including low- and middle-income countries where the incidence of hypoxic-ischemic encephalopathy may be 10-fold higher, with greater rates of neurologic impairment and death. A trial of similar design is ongoing to evaluate this important question (Comparative Outcomes Related to Delivery-Room Cord Milking in Low-Resourced Countries trial; NCT03657394).

Our study focused on 2-year outcomes to evaluate safety and efficacy, but these are not longer term outcomes. Andersson et al^28^ did not find neurodevelopmental benefits with delayed cord clamping compared with ECC at 12 months. but was able to note benefits in fine motor and social domains in boys.^4^ It is possible that the infusion of additional iron and stem cell components of placental blood provided to these infants may have effects not yet seen until early childhood.

## Conclusions

Among nonvigorous infants born at 35 to 42 weeks’ gestation, the parental assessments of neurodevelopmental outcomes at age 2 years were not significantly different comparing UCM and ECC. Considering the important previously reported short-term benefits of UCM to enhance placental transfusion, our study adds further support to this safe, facile, 0-cost intervention as a preferable practice compared with ECC.

## Notes

Supplement 1.
Trial Protocol and Statistical Analysis Plan

Supplement 2. **eTable 1.** Primary and Secondary Outcomes by Randomization Group Among the n = 971 Infants Included in the ASQ-3 Analysis **eTable 2.** Maternal and Neonatal Baseline Characteristics for the Infants Included in the ASQ-3 Analysis (n = 971) vs Those Excluded (n = 759) **eTable 3.** Developmental Screening Characteristics Assessed at 6 and 12 Months by Randomization Group, Including the 7 Infant Deaths (Weighted as Zero)

Supplement 3.
Data Sharing Statement
