# Supplementary material for: Two-Year Outcomes of Umbilical Cord Milking in Nonvigorous Infants: A Secondary Analysis of the MINVI Randomized Clinical Trial
Source: JAMA Netw Open. 2024 Jul 1;7(7):e2416870. doi: 10.1001/jamanetworkopen.2024.16870 (PMC11217871; doi:10.1001/jamanetworkopen.2024.16870)
Supplement: Supplement 1. — Trial Protocol and Statistical Analysis Plan [file jamanetwopen-e2416870-s001.pdf]

Statistical Analysis Plan

Clinical Trials Registration Number: NCT03631940

Updated September 1, 2021

MINVI CONSORT Primary Manuscript Preparation and Analysis Plan

As of September 1, 2021

Table of contents

A. Objectives 2
B. Data preparation 2
C. Manuscript content 2
D. General comments regarding statistics 4
E. Consort diagram 4
F. Descriptive analysis among eligible 6
G. Descriptive analysis among randomized and consented 6
H. Descriptive analysis among randomized and consented 6
I. Primary analysis 8
J. Subgroup analysis 9
K. Sensitivity analyses 9
L. Secondary outcomes 10
M. Adverse events 11
N. Writing Group and Acknowledgments 12

A. Objectives

1) To test whether milking and delayed cord clamping decreases the risk of NICU admission based on predefined criteria compared with early cord clamping in non-vigorous term infants.
2) To test whether milking and delayed cord clamping decreases the risk of secondary safety and efficacy outcomes (use of therapeutic hypothermia, use of volume expanders, hemoglobin, bilirubin levels, death) and exploratory outcomes (length of hospitalization, blood pressure, resuscitation interventions, and HIE) compared with early cord clamping.

B. Data preparation

1) Evaluate all variables, including specify fields - frequencies, distributions, outliers, missing data
   a) DSINFO as an example to output frequencies, etc.

C. Manuscript content

**Title and abstract**<br>• Identification as a randomized trial in the title<br>something like:<br>Cluster-Randomized, Crossover Trial of Umbilical Cord Milking in Non-Vigorous Term Infants<br>or<br>Cluster-Randomized, Crossover Trial of Umbilical Cord Milking and Delayed Clamping versus Early Clamping<br>• Structured summary of trial design, methods, results, and conclusions
**Introduction - background and objectives**<br>• Scientific background and explanation of rationale<br>• Specific objectives or hypotheses
**Methods - trial design and changes to trial design**<br>• Description of trial design (such as parallel, factorial) including allocation ratio<br>• Important changes to methods after trial commencement (such as eligibility criteria), with reasons
**Methods – participants and study settings**<br>• Eligibility criteria for participants<br>• Settings and locations where the data were collected<br>Can include details in a supplement

**Methods - interventions**<br>• The interventions for each group with sufficient details to allow replication, including how and when they were actually administered<br>Can include details in a supplement
**Methods – outcomes and changes to outcomes**<br>• Completely defined pre-specified primary and secondary outcome measures, including how and when they were assessed<br>• Any changes to trial outcomes after the trial commenced, with reasons
**Methods – sample size and interim analyses and stopping guidelines**<br>• How sample size was determined<br>• When applicable, explanation of any interim analyses and stopping guidelines
**Methods – randomization sequence generation and type**<br>**Sequence generation**<br>• Method used to generate the random allocation sequence<br>• Type of randomization; details of any restriction (such as blocking and block size)
**Methods – randomization allocation concealment mechanism**<br>• Mechanism used to implement the random allocation sequence (such as sequentially numbered containers), describing any steps taken to conceal the sequence until interventions were assigned
**Methods – randomization implementation**<br>• Who generated the random allocation sequence, who enrolled participants, and who assigned participants to interventions
**Methods – blinding and similarity of interventions**<br>• If done, who was blinded after assignment to interventions (for example, participants, care providers, those assessing outcomes) and how<br>• If relevant, description of the similarity of interventions<br>Unmasked intervention<br>Investigators masked to study results; blinded adjudication of the primary outcome
**Methods – statistical methods and additional analyses**<br>• Statistical methods used to compare groups for primary and secondary outcomes<br>• Methods for additional analyses, such as subgroup analyses and adjusted analyses
**Results – participant flow and losses and exclusions (figure)**<br>• For each group, the numbers of participants who were randomly assigned, received intended treatment, and were analyzed for the primary outcome<br>• For each group, losses and exclusions after randomization, together with reasons
**Results – recruitment and reason for stopped trial**<br>• Dates defining the periods of recruitment and follow-up<br>• Why the trial ended or was stopped
**Results – baseline data**<br>• A table showing baseline demographic and clinical characteristics for each group
**Results – numbers analyzed**<br>• For each group, number of participants (denominator) included in each analysis and whether the analysis was by original assigned groups
**Results – outcomes and estimation and binary outcomes**<br>• For each primary and secondary outcome, results for each group, and the estimated effect size and its precision (such as 95% confidence interval)<br>• For binary outcomes, presentation of both absolute and relative effect sizes is recommended
**Results – ancillary analyses**<br>• Results of any other analyses performed, including subgroup analyses and adjusted analyses, distinguishing pre-specified from exploratory
**Results – harms**<br>• All important harms or unintended effects in each group
**Discussion - limitations**<br>• Trial limitations, addressing sources of potential bias, imprecision, and, if relevant, multiplicity of analyses

Not masked
**Discussion - generalizability**<br>• Generalizability (external validity, applicability) of the trial findings
**Discussion - interpretation**<br>• Interpretation consistent with results, balancing benefits and harms, and considering other relevant evidence
**Other information – registration**<br>• Registration number and name of trial registry<br>ClinicalTrials.gov Identifier: NCT03631940<br>Umbilical Cord Milking in Non-Vigorous Infants (MINVI)
**Other information - protocol**<br>• Where the full trial protocol can be accessed, if available<br>Supplement
**Other information - funding**<br>• Sources of funding and other support (such as supply of drugs), role of funders<br>Supported by HD096023 from the Eunice Kennedy Shriver National Institute of Child Health and Human Development. The views expressed in this article are those of the authors and do not necessarily represent the views of the National Institutes of Health.

D. General comments regarding statistics

1) The unit of analysis is the neonate.
2) Statistical analyses will be based upon the total cohort of patients randomized into the trial. Patients will be included in the treatment group to which they were randomly assigned regardless of compliance (i.e., intention-to-treat). The primary analysis will include all neonates with primary outcome data.
3) Multivariable analysis will **account for the cluster randomized crossover study design**, with fixed or random effects for treatment group effect, period effect, cluster effect and cluster by period interaction effect. The analysis will also account for multiple gestations and clustering within a pregnancy if more than one neonate of a multi-gestation pregnancy meets eligibility. Analyses accounting only for trial design will be considered unadjusted.
4) If the treatment groups are found to differ on a baseline pre-treatment factor known to be a risk factor for the outcome, the statistical analysis will adjust for these differences (in addition to accounting for trial design). Analyses adjusting for baseline pre-treatment factors will be considered adjusted.
5) An evaluation of treatment by site interaction will be included.

E. Consort diagram

1) Figure 1
2) See example on next page from Anderson et al.

   Anderson CS, Arima H, Lavados P, et al; HeadPoST Investigators and Coordinators. Cluster-Randomized, Crossover Trial of Head Positioning in Acute Stroke. N Engl J Med. 2017;376(25):2437-2447.

   a) Include UAB was randomized and then did not participate
   b) In the example on the next page, remove declined from the first box of exclusions.
   c) Add asterisk to ‘declined to participate’ and footnote with number with waiver of informed consent for primary outcome data.
   d) Add N (%) that had the intervention per protocol, N (%) not per protocol and reason.

      There should not be any with post_rand_excl=yes
   e) Protocol deviations can be included in a figure footnote or in the text only

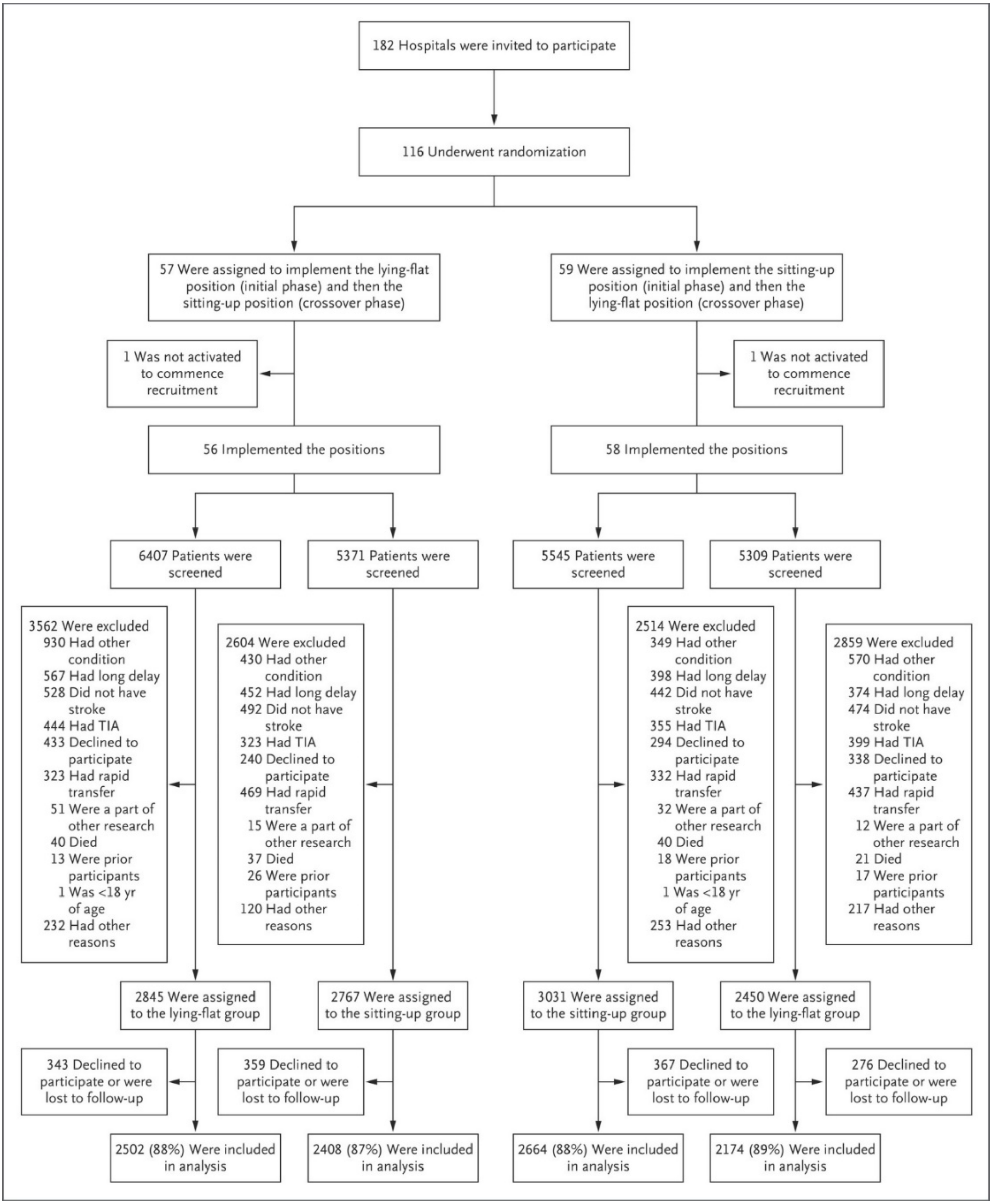

182 Hospitals were invited to participate
116 Underwent randomization
57 Were assigned to implement the lying-flat position (initial phase) and then the sitting-up position (crossover phase)
59 Were assigned to implement the sitting-up position (initial phase) and then the lying-flat position (crossover phase)
1 Was not activated to commence recruitment
1 Was not activated to commence recruitment
56 Implemented the positions
58 Implemented the positions
6407 Patients were screened
5371 Patients were screened
5545 Patients were screened
5309 Patients were screened
3562 Were excluded 930 Had other condition 567 Had long delay 528 Did not have stroke 444 Had TIA 433 Declined to participate 323 Had rapid transfer 51 Were a part of other research 40 Died 13 Were prior participants 1 Was <18 yr of age 232 Had other reasons
2604 Were excluded 430 Had other condition 452 Had long delay 492 Did not have stroke 323 Had TIA 240 Declined to participate 469 Had rapid transfer 15 Were a part of other research 37 Died 26 Were prior participants 120 Had other reasons
2514 Were excluded 349 Had other condition 398 Had long delay 442 Did not have stroke 355 Had TIA 294 Declined to participate 332 Had rapid transfer 32 Were a part of other research 40 Died 18 Were prior participants 1 Was <18 yr of age 253 Had other reasons
2859 Were excluded 570 Had other condition 374 Had long delay 474 Did not have stroke 399 Had TIA 338 Declined to participate 437 Had rapid transfer 12 Were a part of other research 21 Died 17 Were prior participants 217 Had other reasons
2845 Were assigned to the lying-flat group
2767 Were assigned to the sitting-up group
3031 Were assigned to the sitting-up group
2450 Were assigned to the lying-flat group
343 Declined to participate or were lost to follow-up
359 Declined to participate or were lost to follow-up
367 Declined to participate or were lost to follow-up
276 Declined to participate or were lost to follow-up
2502 (88%) Were included in analysis
2408 (87%) Were included in analysis
2664 (88%) Were included in analysis
2174 (89%) Were included in analysis

F. Descriptive analysis among eligible

1) For a supplemental table or text only
2) Comparison of baseline characteristics between consented and non-consented based on all available data (for the non-consented, include those with data obtained under a waiver of informed consent [i.e., minimal dataset])
   a) N (%) and chi square or Fisher's exact for baseline categorical descriptive characteristics
   b) Mean, SD, 25th, median, 75th percentiles and Wilcoxon rank-sum for all baseline continuous descriptive characteristics
3) These results will inform generalizability and potential bias

G. Descriptive analysis among randomized and consented

1) For a supplemental table or text only or just for our information and available if reviewers ask
2) Comparison of baseline characteristics between study periods A and B
   c) N (%) and chi square or Fisher's exact for baseline categorical descriptive characteristics
   d) Mean, SD, 25th, median, 75th percentiles and Wilcoxon rank-sum for all baseline continuous descriptive characteristics
3) These results will inform whether there were imbalances between study period A and B

H. Descriptive analysis among randomized and consented

1) Table 1
2) Comparison of baseline characteristics between treatment groups
   a) N (%) and chi square or Fisher's exact for baseline categorical descriptive characteristics
   b) Mean, SD, 25th, median, 75th percentiles and Wilcoxon rank-sum for all baseline continuous descriptive characteristics
3) These results will describe the study population and inform whether the intervention groups are balanced according to baseline characteristics and which covariables may need to be adjusted for in multivariable analysis. Note: in the multivariable analysis of the primary outcome, some neonates may have data on the primary outcome but may not have data on characteristics.

**Table 1. Characteristics at baseline (pre-intervention)**			
Characteristic<br>confirm whether or not any 2+ of multiples enrolled	Milking and delayed cord clamping<br>(N=)	Early cord clamping<br>(N=)	*P* value<br>(not presented in the manuscript)
**Maternal**			
Race-ethnicity (n, %)<br>**maternal_race maternal_ethnicity**<br>if Hispanic = yes code Hispanic<br>else code per race			
Non-Hispanic White			
Non-Hispanic Black			
Asian			
Hispanic			
Other or unknown			
Age (median, IQR)<br>**maternal_age**			
At least some college education (n, %)<br>**highest_level_of_education**<br>in (5,6,7) (8 is unknown = .)			
Any diabetes (n, %)<br>**mat_diabetes**			

Any hypertension (n, %)<br>**mat_hypertension**
Intrauterine inflammation or infection (n, %)<br>**chorio_triplei**
GBS (n, %)<br>**gbs**
Positive
Negative
Not done
Unknown
Rupture of membranes hours before delivery (median, IQR)<br>**rupture_of_memb**
Intravenous or oral narcotic or CNS depressant medication within 2 hours prior to delivery (n, %)<br>**mat_meds**
General anesthesia (n, %)<br>**general_anesthesia**
Multiple delivery (n, %)<br>**multiple_gestation**
**Neonatal**
Race-ethnicity (n, %)<br>**race ethnicity**<br>if Hispanic = yes code Hispanic<br>else code per race
Non-Hispanic White
Non-Hispanic Black
Asian
Hispanic
Other or unknown
Mode of delivery<br>**mode_of_deliv**<br>code per categories below, but may need to collapse categories; count will be impacted if 2+ of multiple enrolled
Cesarean and assisted (vacuum or forceps)
Cesarean and not assisted
Vaginal and assisted (vacuum or forceps)
Vaginal and not assisted
Gestational age at delivery (median, IQR)<br>**ga_days_mds**
Sex (n, %)<br>**gender**
Male
Female
Ambiguous
Nonvigorous status based on (n, %)<br>**birth_tone breathing_before_cord_mds color_mds**<br>code per categories below, but may need to collapse categories
Color only
Tone only
Breathing only
Color and tone
Color and breathing

Tone and breathing
Color, tone and breathing

Table 2. Delivery room characteristics (immediately peri- or post-intervention)			
Characteristic	Milking and delayed cord clamping (N=)	Early cord clamping (N=)	*P* value (not presented in the manuscript)
Breathing before clamping (n, %) **breathing_before_clamping_mds**			
Apgar score at 1 minute (median, IQR) **minapgar_mds**			
Apgar score at 1 minute <3 (n, %)			
Apgar score at 5 minute (median, IQR) **fiveminapgar_mds**			
Apgar score at 5 minute <7 (n, %)			

I. Primary analysis

1) Table 3
2) Among consented
3) N (%) for primary outcome (NICU admission by predefined criteria) and N (%) for each reason for NICU admission
   a) Primary outcome is NICU admission for any of the following reasons:
      i) Respiratory distress (including any of the following: tachypnea, grunting, retractions)
      ii) Bradycardia/tachycardia
      iii) Hypotonia
      iv) Lethargy/difficult to arouse
      v) Hypertonia/irritability
      vi) Poor feeding/emesis
      vii) Hypoglycemia
      viii) Oxygen desaturations/cyanosis, need for oxygen
      ix) Apnea
      x) Seizures or seizure-like activity
      xi) Hyperbilirubinemia
      xii) Temperature instability
4) In the text, may be good to list any NICU admission reasons that were not included in the primary outcome **not_minvi nonreason ist_other**
5) Relative risk (RR) or odds ratio (OR) and 95% confidence interval for primary outcome for milking vs. early clamping, accounting for the cluster randomized crossover study design, for example, with fixed treatment group effect, fixed period effect, random cluster effect, and random cluster by period interaction effect (hierarchical mixed model regression; PROC GLIMMIX).

Anderson CS, Arima H, Lavados P, et al; HeadPoST Investigators and Coordinators. Cluster-Randomized, Crossover Trial of Head Positioning in Acute Stroke. N Engl J Med. 2017;376(25):2437-2447.

Cameron ST, Glasier A, McDaid L, Radley A, Baraitser P, Stephenson J, Gilson R, Battison C, Cowle K, Forrest M, Goulao B, Johnstone A, Morelli A, Patterson S, McDonald A, Vadiveloo T, Norrie J. Use of effective contraception following provision of the progestogen-only pill for women presenting to community pharmacies for emergency contraception (Bridge-It): a pragmatic cluster-randomised crossover trial. Lancet. 2020 Nov 14;396(10262):1585-1594.

Morgan KE, Forbes AB, Keogh RH, Jairath V, Kahan BC. Choosing appropriate analysis methods for cluster randomized cross-over trials with a binary outcome. Stat Med 2017; 36: 318-33.

Turner RM, White IR, Croudace T. Analysis of cluster randomized cross-over trial data: a comparison of methods. Stat Med 2007; 26: 274-89.

6) The analysis will also account for multiple gestations and clustering within a pregnancy if more than one neonate of a multi-gestation pregnancy was enrolled.
7) If the treatment groups are found to differ on a pre-treatment factor known to be a risk factor for the outcome, the statistical analysis will adjust for these differences.
8) An evaluation of treatment by site interaction will be included as well as an evaluation of adjustment by center to ensure that center differences do not change the conclusion.

Table 3. Primary Outcome

Outcome	Milking and delayed cord clamping (N=)	Early cord clamping (N=)	Relative Risk (95%CI)*	Relative Risk (95%CI)†
NICU admission by predefined criteria make sure to use the adjudicated primary outcome variable **minvi**				
**criteria** can meet more than one criterion; just report n (%), not the RR, for the reasons				
Respiratory distress				
Apnea				
Bradycardia or tachycardia				
Oxygen desaturations or cyanosis, need for oxygen				
Hypotonia				
Lethargy or difficult to arouse				
Hypertonia or irritability				
Hypoglycemia				
Hyperbilirubinemia				
Poor feeding or emesis				
Seizures or seizure-like activity				
Temperature instability				

* accounting for trial design
† accounting for trial design and adjusting for …

J. Subgroup analysis

1) Prior studies offer no basis for assuming *a priori* interactions between any baseline characteristics and treatment group. For this reason, preplanned tests for interactions with treatment assignment are not warranted and are not powered for with the sample size. However, in accordance with NIH guidelines, an evaluation of consistency of the primary outcome across racial-ethnic subgroups will be included.

K. Sensitivity analyses

1) Supplemental table
2) Death is not an anticipated competing risk for the primary outcome as it is expected that all randomized newborns requiring NICU admission will be admitted immediately. However, if any deaths occurred before NICU admission, a sensitivity analysis will be conducted using a composite outcome of NICU admission by predefined criteria or death.
3) Analysis of the primary outcome including those that did not consent but have primary outcome data under a waiver of informed consent.

4) Same as above plus adding counts for those that did not consent and do not have data under a waiver of informed consent, using a range of assumptions.

Sensitivity Analysis				
Outcome and denominator	Milking and delayed cord clamping (N=)	Early cord clamping (N=)	Relative Risk (95%CI)*	Relative Risk (95%CI)†
Primary outcome among consented				
Primary outcome, plus death among consented				
Primary outcome among consented, plus those with outcome data collected under a waiver of informed consent				
Primary outcome among consented, plus those with outcome data collected under a waiver of informed consent, plus assumptions for those without outcome data (0% in milking group; 0% in early group)				
Primary outcome among consented, plus those with outcome data collected under a waiver of informed consent, plus assumptions for those without outcome data (0% in milking group; 25% in early group)				
Primary outcome among consented, plus those with outcome data collected under a waiver of informed consent, plus assumptions for those without outcome data (25% in milking group; 0% in early group)				
Primary outcome among consented, plus those with outcome data collected under a waiver of informed consent, plus assumptions for those without outcome data (25% in milking group; 25% in early group)				
* accounting for trial design<br>† accounting for trial design and adjusting for …				

L. Secondary outcomes

1) Table 2

2) N (%), adjusted RR or OR (95%CI) for dichotomous outcomes (hierarchical mixed model regression); median (IQR), mean adjusted difference for continuous outcomes (general linear mixed models); PROC GLIMMIX or PROC MIXED

3) Secondary safety and efficacy outcomes

   a) Use of therapeutic hypothermia
      cooling

   b) Use of volume expanders
      vol

      i) Also report blood transfusion separately
         bld_tx blood_products

   c) Death through delivery hospitalization

d) Hemoglobin, g/dL, continuous
hgb

e) Peak serum bilirubin, mg/dL, continuous
peak_serum_bilirubin

if missing use transcutaneous bilirubin and add footnote to table
tcbili

ii) Also report phototherapy as a categorical yes/no
recieve_phototh

iii) Also report exchange transfusion for hyperbilirubinemia as a categorical yes/no
hyperbili_transfusion

4) Exploratory outcomes

a) Length of hospitalization, continuous
hosp_days

b) Blood pressure, continuous

Report as mean arterial pressure, calculated from SBP and DBP
adm_bp_yn adm_bp

c) Resuscitation interventions

Multinomial outcome and code the most severe if more than one is checked (some categories may need to be combined). HFNC is not used in the delivery room, only the NICU; would be between supplemental oxygen and CPAP.
1 types_of_support_i___1 None
2 types_of_support_i___2 Supplemental Oxygen
3 types_of_support_i___3 CPAP
4 types_of_support_i___4 PPV (mask or ETT)
5 types_of_support_i___5 Intubation
6 types_of_support_i___6 Compressions
7 types_of_support_i___7 Medications (Epi, volume, other)
types_of_support_i

i) Also report separately maximum fraction of inspired oxygen in delivery room, FiO2, as continuous
max_fio2

d) HIE Defined by the neonatologist examining the infant; we use sarnat scoring. We can grade HIE but the numbers are too small. Need to make sure we didn't miss any mild HIE. (Wade is looking into this to see if there are missed kids.)
sarnat_done sarnat hie

M. Adverse events

1) Table 3 or text only

2) N (%) and chi square or Fisher's exact for all adverse events (that are not primary or secondary outcomes) by treatment group

3) Adverse events – tentative list

a) Neonatal

i) Death through delivery hospitalization

ii) Polycythemia

iii) IVH categories (multinomial)

iv) Pulmonary hemorrhage

b) Maternal

i) Death through delivery hospitalization

N. Writing Group and Acknowledgments

1) Writing group (authors)
2) Investigators and other study personnel
