# Supplementary material for: Two-Year Outcomes of Umbilical Cord Milking in Nonvigorous Infants: A Secondary Analysis of the MINVI Randomized Clinical Trial
Source: JAMA Netw Open. 2024 Jul 1;7(7):e2416870. doi: 10.1001/jamanetworkopen.2024.16870 (PMC11217871; doi:10.1001/jamanetworkopen.2024.16870)
Supplement: Supplement 2. — eTable 1. Primary and Secondary Outcomes by Randomization Group Among the n = 971 Infants Included in the ASQ-3 Analysis eTable 2. Maternal and Neonatal Baseline Characteristics for the Infants Included in the ASQ-3 Analysis (n = 971) vs Those Excluded (n = 759) eTable 3. Developmental Screening Characteristics Assessed at 6 and 12 Months by Randomization Group, Including the 7 Infant Deaths (Weighted as Zero) [file jamanetwopen-e2416870-s002.pdf]

Supplementary Online Content

Katheria AC, El Ghormli L, Clark E, et al. Two-year outcomes in the umbilical cord milking in nonvigorous infants: a secondary analysis of the MINVI randomized clinical trial. *JAMA Netw Open*. 2024;7(6):e2416870. doi:10.1001/jamanetworkopen.2024.16870

eTable 1. Primary and Secondary Outcomes by Randomization Group Among the n=971 Infants Included in the ASQ-3 Analysis

eTable 2. Maternal and Neonatal Baseline Characteristics for the Infants Included in the ASQ-3 Analysis (n=971) vs Those Excluded (n=759)

eTable 3. Developmental Screening Characteristics Assessed at 6 and 12 Months by Randomization Group, Including the 7 Infant Deaths (Weighted as Zero)

This supplementary material has been provided by the authors to give readers additional information about their work.

© 2024 Katheria AC et al. *JAMA Network Open.*

eTable 1. Primary and Secondary Outcomes by Randomization Group among the n=971 Infants Included in the ASQ-3 Analysis

Characteristic	Umbilical Cord Milking (n=502)	Early Cord Clamping (n=469)	P-value
**Primary Outcome**			
[a]NICU admission by predefined criteria	135 (27)	129 (28)	0.83
**Secondary Outcomes**			
[b]Cardiorespiratory support	298 (59)	331 (70)	<0.01**
[c]Therapeutic hypothermia	20 (4)	27 (6)	0.20
Volume bolus (i.e., normal saline)	44 (9)	42 (9)	0.92
Phototherapy	76 (15)	59 (12)	0.25
Hypoxic-ischemic encephalopathy			
Any (mild/moderate/severe)	23 (5)	29 (6)	0.27
Moderate-severe	11 (2)	20 (4)	0.07
[d]Serious adverse event	1 (<1)	5 (1)	0.08
Apgar score at 1 minute ≤3	147 (29)	165 (35)	0.05
Apgar score at 5 minutes ≤6	75 (15)	86 (18)	0.16

Data are n (%). P-value from a Chi-square test evaluating differences between the two groups. Additional analyses accounting for the clustered-crossover study design provided similar to the unadjusted analyses shown in the above table.
[a]Defined as admission to the NICU in the first 24 hours of life for predefined criteria: respiratory distress (tachypnea, grunting, retractions), bradycardia or tachycardia, hypotonia, lethargy or difficult to arouse, hypertonia or irritability, poor feeding or emesis, hypoglycemia, oxygen desaturations or cyanosis, need for oxygen, apnea, seizures or seizure-like activity, hyperbilirubinemia, and/or temperature instability. More than one criterion could be met.
[b]Includes supplemental oxygen, continuous positive airway pressure, positive pressure, ventilation, intubation, compressions, and medications (e.g., epinephrine, volume)
[c]P-value is no longer significant in a model accounting for the clustered-crossover study design (p=0.09)
[d]Infants with mild hypoxic-ischemic encephalopathy at some centers received therapeutic hypothermia.
[e]Includes death before discharge, polycythemia, hyperbilirubinemia, requiring an exchange transfusion, severe intraventricular hemorrhage, and pulmonary hemorrhage.

© 2024 Katheria AC et al. *JAMA Network Open.*

eTable 2. Maternal and Neonatal Baseline Characteristics for the Infants Included in the ASQ-3 Analysis (n=971) vs those Excluded (n=759)

Characteristic	Included in the Analysis (n=971)	Excluded from the Analysis (n=759)	P-value
**Maternal**			
[b]Race-ethnicity			<.001
Hispanic	174 (18)	59 (8)	
Non-Hispanic Asian	84 (9)	21 (3)	
Non-Hispanic Black	68 (7)	40 (5)	
Non-Hispanic White	544 (56)	97 (13)	
Other/Unknown	101 (10)	542 (71)	
Age, yr.	31 (27-35)	29 (25-33)	<.001
At least some college education	670/852 (78)	118/192 (61)	<.001
Any diabetes	135 (14)	63 (12)	.38
Any hypertension	170 (17)	102 (20)	.26
Intrauterine inflammation or infection	116 (12)	64 (12)	.78
GBS positive	202/970 (21)	101/514 (20)	.21
Rupture of membranes before delivery, hr.	6 (1-14)	6 (1-14)	.56
[a]Narcotic/CNS depressant ≤ 2 hrs. pre-delivery	102 (10)	27 (11)	.88
General anesthesia	65/970 (7)	17/248 (7)	.93
**Neonatal**			
Female sex	425 (44)	305 (45)	.55
Cesarean mode of delivery	452 (46)	231 (30)	<.001
Multiple gestation	31/968 (3)	19/514 (4)	.62
Gestational age at delivery, wk.	39 (38-40)	39 (38-40)	.69

Data are n (%), or median (IQR), unless otherwise noted. P-value from a Wilcoxon test or Chi-square test evaluating differences between the two groups. Denominators for all variables are as per column headers unless otherwise noted.
GBS = Group B Streptococcus; CNS = central nervous system
[a]Oral or intravenous administration
[b]Maternal race/ethnicity not available among 71% of the newborns excluded from analysis.

© 2024 Katheria AC et al. *JAMA Network Open.*

eTable 3. Developmental Screening Characteristics Assessed at 6 and 12 Months by Randomization Group, Including the 7 Infant Deaths (Weighted as Zero)

	**6 Months (N=818)**		**12 Months (N=782)**	
	Umbilical Cord Milking (n=415)	Early Cord Clamping (n=403)	Umbilical Cord Milking (n=403)	Early Cord Clamping (n=372)
**ASQ-3 Domains**				
Communication	50 (45-55)	50 (45-55)	50 (40-60)	50 (40-55)
Gross Motor	45 (35-55)	45 (35-50)	55 (45-60)	50 (40-60)
Fine Motor	55 (40-60)	50 (40-60)	55 (45-60)	55 (50-60)
Problem Solving	55 (45-60)	55 (45-60)	50 (40-55)	50 (40-55)
Personal-Social	50 (40-60)	50 (40-55)	45 (35-55)	45 (40-55)
Overall score	250 (220-270)	245 (217-270)	245 (215-275)	245 (215-270)

Data are or median (IQR).

© 2024 Katheria AC et al. *JAMA Network Open.*
