# Supplementary material for: Two-Year Outcomes of Umbilical Cord Milking in Nonvigorous Infants: A Secondary Analysis of the MINVI Randomized Clinical Trial
Source: JAMA Netw Open. 2024 Jul 1;7(7):e2416870. doi: 10.1001/jamanetworkopen.2024.16870 (PMC11217871; doi:10.1001/jamanetworkopen.2024.16870)
Supplement: Supplement 3. — Data Sharing Statement [file jamanetwopen-e2416870-s003.pdf]

Data Sharing Statement

Katheria. Two-Year Outcomes of Umbilical Cord Milking in Nonvigorous Infants. *JAMA Netw Open*. Published June 20, 2024. doi:10.1001/jamanetworkopen.2024.16870

Data
Data available: No

Additional Information
Explanation for why data not available: Will need to have requests submitted prior to approval
